# Effect of Photobiomodulation on Post-Endodontic Pain Following Single-Visit Treatment: A Randomized Double-Blind Clinical Trial

**DOI:** 10.3390/jpm15080347

**Published:** 2025-08-02

**Authors:** Glaucia Gonçales Abud Machado, Giovanna Fontgalland Ferreira, Erika da Silva Mello, Ellen Sayuri Ando-Suguimoto, Vinicius Leão Roncolato, Marcia Regina Cabral Oliveira, Janainy Altrão Tognini, Adriana Fernandes Paisano, Cleber Pinto Camacho, Sandra Kalil Bussadori, Lara Jansiski Motta, Cinthya Cosme Gutierrez Duran, Raquel Agnelli Mesquita-Ferrari, Kristianne Porta Santos Fernandes, Anna Carolina Ratto Tempestini Horliana

**Affiliations:** 1 Biophotonics-Medicine Postgraduate Program, UNIVERSIDSADE NOVE DE JUHO (UNINOVE), São Paulo 01525-000, Brazil; glaugoncales@gmail.com (G.G.A.M.); giovannafontgallandf@gmail.com (G.F.F.); erikasmello@gmail.com (E.d.S.M.); esa2406@gmail.com (E.S.A.-S.); marcia-cabral@uni9.pro.br (M.R.C.O.); janainyaltrao@gmail.com (J.A.T.); apaisano@uni9.pro.br (A.F.P.); camacho@uni9.pro.br (C.P.C.); sandra.skb@gmail.com (S.K.B.); larajmotta@uni9.pro.br (L.J.M.); cduran@uni9.pro.br (C.C.G.D.); raquel.mesquita@gmail.com (R.A.M.-F.); kristianneporta@gmail.com (K.P.S.F.); 2 Undergraduate Program in Dentistry, Universidade Nove de Julho, UNINOVE, São Paulo 01525-000, Brazil; viniciusleaoroncolatoo@hotmail.com; 3 Postgraduate Program in Medicine, Universidade Nove de Julho, UNINOVE, São Paulo 01525-000, Brazil; 4 Postgraduate Program in Rehabilitation Sciences, Universidade Nove de Julho, Uninove, São Paulo 01525-000, Brazil

**Keywords:** endodontics, low-level light therapy, pain management, periapical periodontitis, photobiomodulation

## Abstract

The evidence for photobiomodulation in reducing postoperative pain after endodontic instrumentation is classified as low or very low certainty, indicating a need for further research. Longitudinal pain assessments over 24 h are crucial, and studies should explore these pain periods. **Background/Objectives**: This double-blind, randomized controlled clinical trial evaluated the effect of PBM on pain following single-visit endodontic treatment of maxillary molars at 4, 8, 12, and 24 h. Primary outcomes included pain at 24 h; secondary outcomes included pain at 4, 8, and 12 h, pain during palpation/percussion, OHIP-14 analysis, and frequencies of pain. **Methods**: Approved by the Research Ethics Committee (5.598.290) and registered in Clinical Trials (NCT06253767), the study recruited adults (21–70 years) requiring endodontic treatment in maxillary molars. Fifty-eight molars were randomly assigned to two groups: the PBM Group (*n* = 29), receiving conventional endodontic treatment with PBM (100 mW, 333 mW/cm^2^, 9 J distributed at 3 points near root apices), and the control group (*n* = 29), receiving conventional treatment with PBM simulation. Pain was assessed using the Visual Analog Scale. **Results**: Statistical analyses used chi-square and Mann–Whitney tests, with explained variance (η^2^). Ten participants were excluded, leaving 48 patients for analysis. No significant differences were observed in postoperative pain at 24, 4, 8, or 12 h, or in palpation/percussion or OHIP-14 scores. Pain frequencies ranged from 12.5% to 25%. **Conclusions**: PBM does not influence post-treatment pain in maxillary molars under these conditions. These results emphasize the importance of relying on well-designed clinical trials to guide treatment decisions, and future research should focus on personalized dosimetry adapted to the anatomical characteristics of the treated dental region to enhance the accuracy and efficacy of therapeutic protocols.

## 1. Introduction

Apical periodontitis is one of the most prevalent inflammatory oral diseases worldwide, with 50% of the adult population having at least one compromised tooth [1,2]. Endodontic treatment primarily addresses apical periodontitis [1,3]. Post-instrumentation pain remains challenging despite significant technological advances, such as rotary and reciprocating instruments [4], which is likely linked to the increased release of C-type nerve fiber neuropeptides, potentially triggered by the extrusion of infected debris into the periapical region [3]. High pain levels can impair a patient’s quality of life and compromise treatment outcomes [5].

Postoperative pain following endodontic intervention is common, with prevalence rates between 3% and 58%, especially within the first 24 h, decreasing significantly by the seventh day [6]. Elevated levels of inflammatory mediators activate or sensitize peripheral nociceptors, causing peripheral hyperalgesia [5,7]. Chemical, mechanical, or microbial lesions in the pulp and periapical tissues increase the expression of neuropeptides from type C nerve fibers, contributing to peripheral inflammation [5,8].

Pharmacological treatment, especially with NSAIDs, is the first choice for pain management. However, caution is needed in patients with high cardiovascular risk, as lower doses and shorter treatment durations are recommended for safety and effectiveness [9]. This limitation underscores the need for alternative therapies for those unable to safely use NSAIDs. Ethical considerations in initial research often prioritize healthy populations [10], favoring individuals with fewer health risks over those with contraindications, such as cardiovascular conditions. This trial aims to assess the effect of sole PBM on pain following single-session endodontic instrumentation of maxillary molars.

Photobiomodulation therapy (PBM) has been considered an adjunct to endodontic techniques to prevent or reduce post-endodontic pain [11]. As a non-invasive, cost-effective, and easy-to-administer method, PBM is promising for patients who do not tolerate NSAIDs well [12].

Irradiation with photons in the red to near-infrared light spectrum can induce anti-inflammatory effects by inhibiting Prostaglandin E2 (PGE2) production and mRNA expression of cyclooxygenases (COX1 and COX2). Irradiation at these wavelengths reduces reactive oxygen species (ROS) that mediate calcium-dependent Phospholipase A2 (FLA2) expression, secretory FLA2 (sPLA2), and COX2 and inhibits PGE2 release [12].

Randomized clinical trials have demonstrated that PBM results in lower pain levels for patients undergoing endodontic treatment compared to control groups, especially in the early postoperative period [7,8,13]. However, the evidence is low to very low, with a lack of standardization [4,8,14,15,16].

Studies are needed to standardize dental samples (e.g., restricting the sample to molars only), dosimetric parameters, control of medication use, and longitudinal postoperative pain assessments at critical 24 h intervals. Furthermore, all published studies have focused on mandibular elements, with no protocols established for photobiomodulation in maxillary elements. Given these considerations, this trial aims to evaluate the effect of PBM on pain following single-session endodontic instrumentation of maxillary molars at 4, 8, 12, and 24 h.

## 2. Materials and Methods

**Figure 1:**
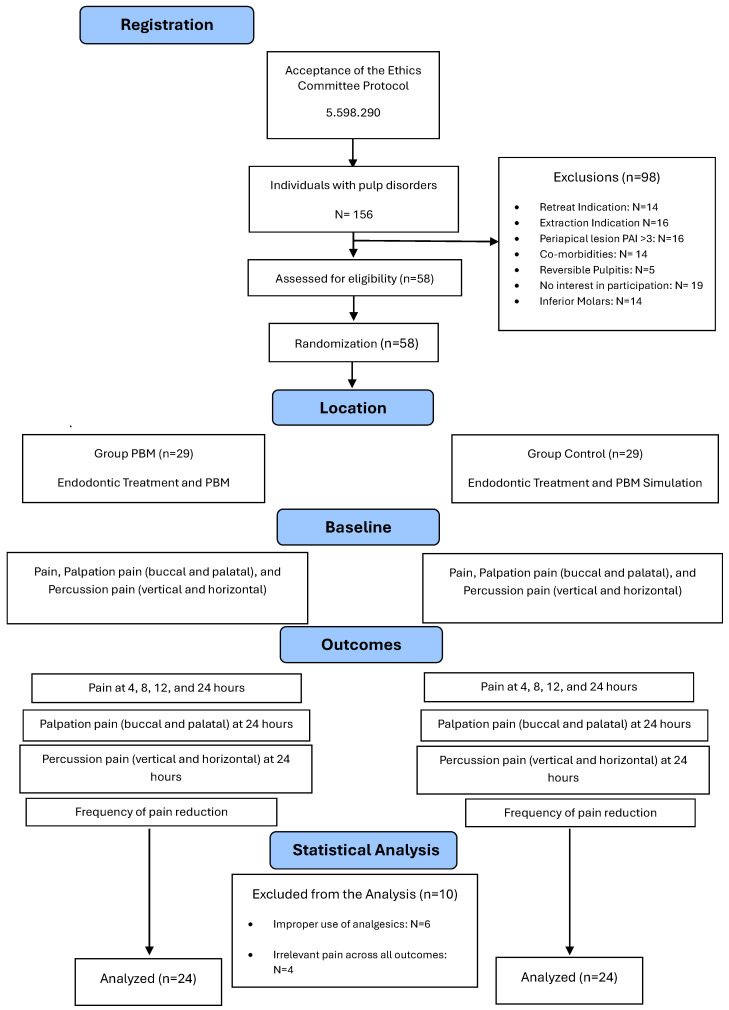
Flowchart of the study by Consort, 2010.

This randomized double-blinded controlled clinical trial has been written according to CONSORT guidelines and explanation [17] and was structured as the flowchart (Figure 1). The Research Ethics Committee approved this study on 23 August 2022 (https://plataformabrasil.saude.gov.br, accessed on 20 June 2022), number: 5.598.290. The project was also registered on ClinicalTrials (https://clinicaltrials.gov/, accessed on 1 March 2023), NCT06253767.

Participants signed the informed consent form after receiving verbal and written study explanations. The study adhered to the Helsinki Declaration. This trial was performed from October 2022 to June 2024 at the university’s dental clinic, the sole research center. The experimental design was a double-blinded, randomized clinical trial with a two-arm, parallel design (1:1 allocation ratio).

All endodontic treatments and pain assessments were performed by a single operator blinded to the random allocation of participants.

### 2.1. Sample Size Calculation

The sample size was calculated based on Naseri et al., 2020 [7], which evaluated post-endodontic treatment pain over 24 h with PBM application. Using GPower 3.1 software for *t*-tests with a two-tailed hypothesis, a significance level of 5%, and a power of 95%, an estimated 48 patients were needed, with 24 participants per group. To account for a 20% follow-up loss, 58 participants were included, with 29 in each group.

### 2.2. Inclusion/Exclusion Criteria

Participants included a diagnosis of asymptomatic irreversible pulpitis and a periapical diagnosis of chronic apical periodontitis without apparent rupture of the lamina dura on radiographic examination in three-rooted elements (maxillary molars), aged between 21 and 70 years of both genders. The absence of clinically relevant spontaneous pain was eligible for inclusion. Exclusion criteria were any conditions or factors that alter pain perception, increase the treatment time of a single session to more than 90 min, or prevent the procedure from being performed, such as: pregnancy or breastfeeding, oncological or renal diseases, diabetes, drug, alcohol or tobacco use, immunosuppression, use of medications (such as analgesics, NSAIDs, corticosteroids or antibiotics at least ten days before treatment), any periodontal disease (periodontitis and gingivitis), anatomical variations that would compromise or extend single-visit treatment beyond 90 min, previous endodontic treatment, asymptomatic non-microbial apical periodontitis, extensive coronary involvement, or severe allergy to chlorine.

### 2.3. Randomization

Participants were randomly assigned to experimental groups using a list generated by a researcher not involved in the study (https://www.sealedenvelope.com/, accessed on 10 August 2022). Group allocation was 1:1. Sequentially numbered opaque envelopes contained the treatment assignment and were maintained in numerical order until treatment administration.

### 2.4. Experimental Design

Participants were divided into two groups.

**Figure 2:**
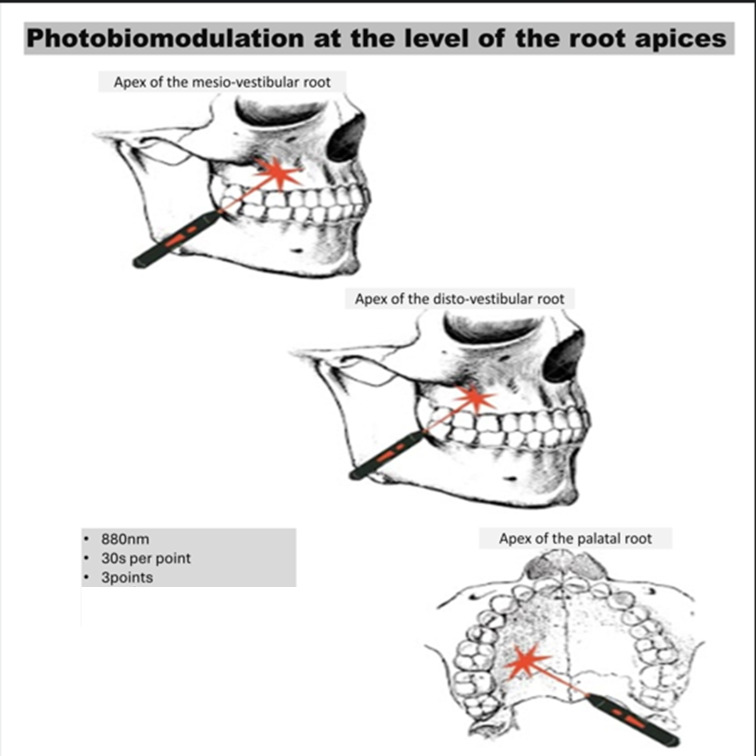
Abstract Image—PBM. Source: Author (original illustration).

PBM Group (*n* = 29): Received standard endodontic treatment with photobiomodulation with protocols adapted from Lopes et al., 2019 [13], using 808 nm (AsGaAl) infrared irradiation from Laser Duo^®^ equipment (MMOptics^®^, São Paulo, Brazil), delivering 9 J distributed at three points per root apex (Table 1; Figure 2).

Control Group (*n* = 29): Received standard endodontic treatment with simulated PBM application exactly as the PBM Group. All safety preparations were conducted (including the placement of protective eyewear), and equipment sounds were simulated without activation.

### 2.5. Study Blinding

The principal researcher, an endodontist (GGAM), conducted initial pain analyses and standard endodontic treatment without knowledge of group assignments. A second researcher (VLR), duly qualified to apply photobiomodulation, performed the PBM application based on randomization. This researcher was the only one aware of treatment allocation and was not involved in outcome analysis. Participants were blinded to the treatment, all preparations were conducted, and equipment sounds were simulated without activation.

### 2.6. Pre-Treatment Assessments and Endodontic Diagnosis

The individuals signed the Informed Consent Form and underwent a medical history review and initial radiographic examination. The medical history consisted of questions about the participant’s overall health, demographic data (age, gender), and medical history.

A thermal test was also performed, and the pulp response was recorded in the patient’s clinical chart. Dental elements were selected with a diagnosis of asymptomatic irreversible pulpitis and a periapical diagnosis of chronic apical periodontitis without apparent rupture of the lamina dura on radiographic examination.

### 2.7. Baseline

The baseline assessment for pain, pain on buccal palpation, pain on palatal palpation, pain on vertical percussion, and pain on horizontal percussion was conducted using the Visual Analog Scale (VAS), completed by the patient before endodontic treatment and after palpation and percussion procedures. The VAS consisted of a 10 cm line, with “0” indicating no pain and “10” indicating maximum pain. The patient marked the scale for each parameter, and the distance from zero to the mark was measured with a millimeter ruler. The values were then recorded in the clinical chart.

### 2.8. Endodontic Technique

All participants underwent standard endodontic treatment by a single operator (principal researcher), receiving gold-standard treatment proposed for the condition. They were anesthetized with one cartridge of 2% mepivacaine (20 mg/mL) with 1:100,000 epinephrine (0.01 mg/mL) (DFL^®^, Rio de Janeiro, Brazil). Following anesthesia, absolute isolation was achieved using a rubber dam (Angelus^®^, Londrina, Brazil) and a gingival barrier (Top Dam^®^ FGM Dental, FGM^®^, Joinville, Brazil) for optimal sealing. The involved clinical dental crown was disinfected with 2% chlorhexidine (Formula e Ação^®^, São Paulo, Brazil), and the coronal access was performed.

The endodontic treatment proceeded with the following steps: Preparation of the coronal part of the root canal was performed using rotary Pre-Race files (FKG Dentaire^®^, La Chaux-de-Fonds, Switzerland) 35.08 at 600 rpm and torque of 3N with a VDW Silver motor (VDW^®^, Munich, Germany), followed by instrumentation of the middle thirds using rotary instruments of taper 10.02, 15.02, and 20.02 (FKG Dentaire^®^, La Chaux-de-Fonds, Switzerland) for patency.

An apical locator APX1 (GNATUS^®^, Ribeirão Preto, Brazil) was used to determine the tooth length, with 1 mm subtracted for working length determination. Canal reshaping was conducted using reciprocating WaveOne Gold (DENTSPLY^®^, York, PA, USA) with sizes 20.07 and 25.07, supplemented with 35.06 (as needed) on the VDW Silver motor (VDW^®^, Munich, Germany) set to the established WaveOne function. After each file change, abundant irrigation with Endo-Eze 1′′ Irrigator tips (Ultradent Products, Inc.^®^, South Jordan, UT, USA) and 2.5% sodium hypochlorite (Asfer^®^, São Paulo, Brazil) was performed. The total volume of irrigation through the instrumentation was standardized to 50 mL of 2.5% sodium hypochlorite.

After completion of chemical-mechanical canal preparation and shaping, the final irrigation protocol using Easy Clean (Bassi^®^, Belo Horizonte, MG, Brazil) with rotation between 10,000 and 15,000 r.p.m. in 3 agitation cycles of 20 s each with 2.5% sodium hypochlorite, followed by 3 agitation cycles of 20 s each with 17% EDTA (Fórmula e Ação^®^, São Paulo, Brazil), and final irrigation with 2.5% sodium hypochlorite (Asfer^®^, São Paulo, Brazil).

The canals were dried using capillary tips (Ultradent Products, Inc.^®^, South Jordan, UT, USA) for suction, followed by sterile absorbent paper points (DENTSPLY^®^, York, PA, USA). Obturation using the single cone technique matched to the final file size (DENTSPLY^®^, York, PA, USA) used for root filling. A traditional lateral condensation technique with digital spreaders (DENTSPLY^®^, York, PA, USA) was necessary until the canal filling was complete. The obturation stage was followed by restoration of the coronal portion (3M ESPE^®^, St. Paul, MN, USA).

The elements’ occlusion was thoroughly assessed and adjusted as necessary, preventing premature contact.

### 2.9. Outcomes

#### 2.9.1. Pain at 24 H (Primary Outcome)

Pain levels were assessed using the Visual Analog Scale (VAS) at baseline and 24 h post-treatment. The VAS is a 10 cm line where “0” represents no pain and “10” represents maximum pain. Participants marked their pain level on the line, and the distance from zero to the mark was measured in millimeters.

#### 2.9.2. Pain (Secondary Outcome) at 4, 8, and 12 H Post-Treatment

Participants were provided with a pain assessment sheet to document pain levels at 4, 8, and 12 h after the procedure. Telephone contacts were made during these intervals to ensure the accurate completion of the forms, and participants returned the completed sheets to the principal investigator at the 24 h follow-up.

#### 2.9.3. Palpation Pain at 24 H (Secondary Outcome)

Palpation pain was assessed by applying gentle digital pressure to the apical region of the treated tooth, both vestibular and palatal, and measured using the VAS.

#### 2.9.4. Percussion Pain at 24 H (Secondary Outcome)

▪ Horizontal percussion: The tooth was pressed against the lateral alveolar walls using a number 5 mirror handle, maintaining perpendicular contact with the buccal surface.
▪ Vertical percussion: The tooth was pressed against the alveolar base using the mirror handle. Pain was measured with the VAS.

#### 2.9.5. Analysis of Analgesic Medication (Secondary Outcome)

Following the completion of endodontic treatment, all participants were provided with 750 mg paracetamol tablets [7]. Paracetamol 750 mg is an analgesic known to provide significant postoperative pain relief [9]. Participants were instructed to mark the 10 cm on the Visual Analog Scale (VAS) if they experienced maximum pain (10 cm VAS) and to take the medication accordingly, recording the time of intake.

Only six participants (four from the control group and two from the PBM group) reported taking the prescribed analgesic. However, their intake was inappropriate, as they used it preventively due to fear of pain. As a result, these participants were excluded from statistical analysis, and this outcome was not considered.

#### 2.9.6. Analysis of the OHIP-14

The effects of treatment on participants’ quality of life were evaluated using the patient-centered outcome measures from the Oral Health Impact Profile-14 (OHIP-14). Participants respond to 14 questions, scoring each answer on a scale of 0 (never), 1 (rarely), 2 (sometimes), 3 (most of the time), and 4 (always). Using a direct analysis method, scores are summed (0–56), with 56 indicating the highest impact on quality of life [18]. The questionnaire was given to participants at the end of treatment, with thorough instructions on how to complete it and the importance of submitting it at the 24 h follow-up post-treatment.

#### 2.9.7. Analysis of the Frequencies

In this study, the frequency of pain was analyzed at 4, 8, 12, and 24 h after endodontic instrumentation in single-visit treatment of maxillary molars, based on the Youden index.

### 2.10. Statistical Analysis

Categorical variables were compared using chi-square or likelihood ratio tests. Quantitative variables were assessed for normality using the Kolmogorov–Smirnov test. Age was reported as mean and standard deviation, while other variables were presented as median and interquartile range (IQR).

The Student’s *t*-test was used to analyze age differences between groups. In contrast, Pearson’s chi-square test was used to analyze tooth type (first and second maxillary molars) and the proportion of female participants between groups. Inter-group comparisons were performed using the Mann–Whitney U test.

The frequency of pain was calculated by the ratio of cases showing pain at 4, 8, 12, and 24 h to the total number of cases.

An association analysis was performed between the variables Group (PBM or Control) and the categorization of outcomes based on the Youden index. Subsequently, the explained proportional variance analysis, PVE (η ^2^), was calculated.

All analyses were conducted using SPSS 29 (released in 2023; IBM SPSS Statistics for Windows, Version 29.0.2.0. Armonk, NY: IBM Corp.), with significance set at *p* < 0.05.

The dataset analysis for area under the curve was conducted using Scientific Python Development Environment (Spyder v6.0.7) [19]. Data processing was performed with Pandas (v2.0.1) and NumPy(v1.26.0) [20,21]. SciPy (v1.16.1) was used for statistical analysis [22]. Graphical visualizations were generated with Seaborn (v0.13.2) and Matplotlib (v3.6.3) [23,24].

## 3. Results

Fifty-eight participants were randomized. However, six patients were excluded for preemptively using analgesics and four for not experiencing initial or postoperative pain in any analyzed parameters. A total of 48 participants were available for the final analysis, with 24 participants in each group.

### 3.1. Demographic Data

The groups were homogeneous in terms of demographic and clinical variables. The mean age and standard deviation were 41.83 ± 10.54 years for the PBM group and 40.75 ± 13.310 years for the control group (*p* = 0.756). The proportions of females were similar between the groups (50% vs. 70.84%, *p* = 0.140), as were the types of treated teeth (first or second molar) (*p* = 0.074) (Table 2).

### 3.2. Primary and Secondary Outcomes

Outcomes were assessed at various times, and no significant differences were observed between groups for pain at 24 h (*p* = 0.515), 4 h (*p* = 0.774), 8 h (*p* = 0.887), and 12 h (*p* = 0.772). Similarly, for complementary examinations of palpation and percussion, no differences were noted between the groups at the 24 h intervals for vestibular palpation (*p* = 0.651), palatal palpation (*p* = 0.686), vertical percussion (*p* = 0.704), and horizontal percussion (*p* = 0.974) (Table 3).

The explained proportional variance analysis (PVE) showed the highest value for the primary outcome of pain at 24 h (η^2^ = 0.160) and the lowest for pain at 12 h (η^2^ = 0.000).

**Figure 3:**
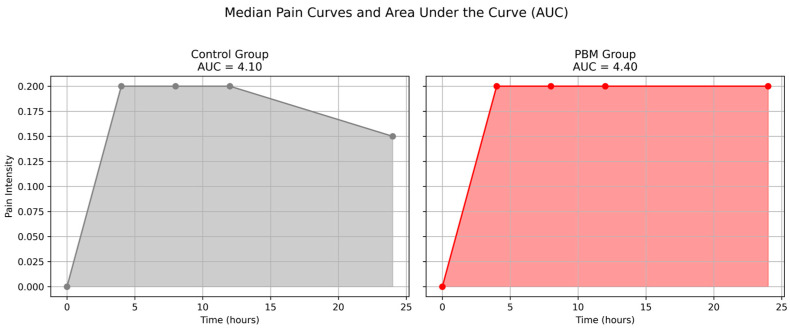
Median pain curves and area under the curve. Source: Author.

Figure 3 illustrates the median pain intensity curves over a 24 h period for both the control and PBM groups, along with their respective area under the curve (AUC) values. Both groups exhibited a rapid increase in pain intensity within the first 4 h following endodontic treatment. In the control group, pain intensity remained stable until approximately 12 h, followed by a gradual decline up to 24 h (AUC = 4.10). In contrast, the PBM group showed a sustained plateau in pain intensity from 4 to 24 h, with a slightly higher AUC (4.40). However, Mann–Whitney test results indicated no statistically significant difference between groups (*p* = 0.695; η^2^ = 0.05) for the AUC comparison.

**Figure 4:**
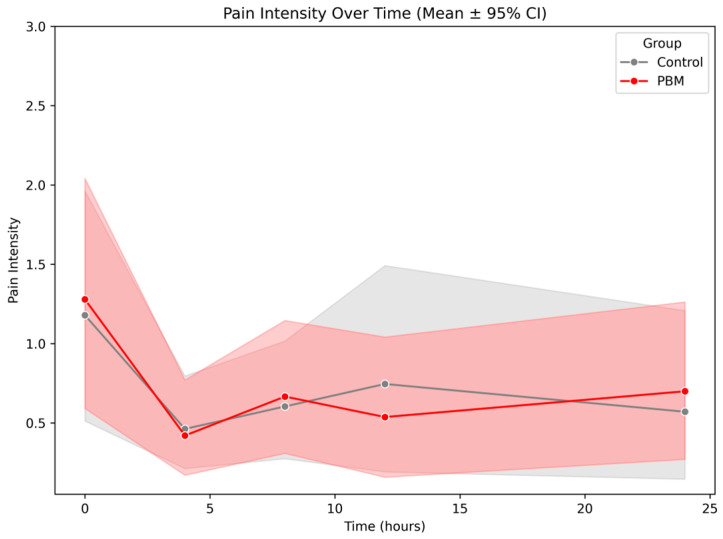
Pain intensity over time. Source: Author.

Figure 4 shows the mean pain intensity over time (±95% confidence interval) for the control and PBM groups over a 24 h period. Both groups started with similar pain levels immediately following the endodontic procedure. The PBM group consistently exhibited slightly lower mean pain scores at all time points; however, the substantial overlap in confidence intervals between groups indicates a lack of statistically significant difference.

Similarly, no statistically significant difference was found between the groups (*p* = 0.347) for the OHIP-14 outcome regarding the effects of treatment on participants’ quality of life (Table 4).

Pain frequencies were calculated for all time intervals analyzed based on the Youden index. No differences were found between the groups for the 4 h (*p* = 0.477), 8 h (*p* = 0.731), 12 h (*p* = 1.000), and 24 h (*p* = 0.267) intervals (Table 5).

## 4. Discussion

This randomized, double-blind clinical trial evaluated the effect of photobiomodulation (PBM) on postoperative pain following single-session endodontic treatment of maxillary molars. Contrary to the initial hypothesis that PBM would reduce postoperative pain levels, no significant differences were observed between the PBM and control groups at any of the evaluated time intervals (4, 8, 12, and 24 h) or during the semiological assessments of palpation and percussion.

Systematic reviews have highlighted the need for standardization in dosimetric parameters, sample selection, medication control, and pain assessment within the critical 24 h period after treatment [3,5,14,15]. In line with these recommendations, this study employed a clinically validated PBM dosimetry protocol. It adhered to a rigorous randomized controlled trial (RCT) design, ensuring standardized medication use (only paracetamol) and a homogeneous sample consisting solely of maxillary molars diagnosed with asymptomatic irreversible pulpitis and chronic apical periodontitis.

The study was carefully designed to control variables that could influence the results, such as the operator’s skill and the exclusion of patients using analgesics or with conditions that might affect pain perception. Initially, the focus was on maxillary molars, believing that the pneumatic nature of the maxilla, with its lower bone density and reduced hydroxyapatite content compared to the mandible, would allow greater light diffusion and penetration [25]. However, the expected therapeutic improvement was not observed. No previous study has specifically focused on maxillary molars in this context, and all prior clinical studies were conducted on mandibular molars [7,8,11,13,26,27,28,29].

While the study encompassed a broad age range, which could potentially introduce variability, the statistical analysis revealed no significant differences between groups for age (*p* = 0.756), gender (*p* = 0.140), and treated tooth (*p* = 0.074). The mean ages were comparable, approximately 40 years in both the control (40.75 ± 13.31) and treatment (41.83 ± 10.54) groups, indicating homogeneity in age distribution across groups.

Although the maxilla is not a perfectly transparent structure, its lower hydroxyapatite content suggests that light diffusion should be more effective than in the mandible. Unfortunately, our results indicate that this anatomical difference did not translate into better therapeutic outcomes with PBM. Despite efforts, the absence of observed differences raises questions about the efficacy of PBM in pain control after endodontic treatment. A plausible explanation for the lack of significant results may be related to the limitations of infrared light penetration through the anatomical structures of maxillary molars, particularly the palatal root.

Bone thickness between the buccal and palatal/lingual cortical plates in both the maxilla and mandible has been shown to vary significantly, depending on individual anatomical biotypes [30]. Although this difference generally does not exceed 5 mm, the bone structure, along with the inherent dispersion of light within biological tissues, may prevent effective photon energy delivery to deeper regions [7], such as the palatal root of maxillary molars.

The impact of tissue penetration depths by PBM in endodontics still lacks scientific evidence [5]. While there is an understanding of the cellular effects of PBM, uncertainties remain regarding the impact of these modifications on reducing post-endodontic pain [3,14].

Supporting this hypothesis, studies demonstrated that near-infrared light penetration is significantly limited in thicker bone tissues, resulting in insufficient fluence reaching the target tissue to activate the desired biological processes [31]. This limitation may explain the lack of therapeutic effects observed in the PBM group in the present study, as light failed to adequately penetrate the dense bone structure around the roots of maxillary molars, particularly the palatal root. Future studies should consider including an analysis of the initial bone thickness through tomographic scans (CBCT) of each participant’s apical region.

Another important parameter to consider when evaluating whether light reaches the target is irradiance, defined as the ratio of the average incident power to the output area of the device or the area incident on the treated surface [32]. Due to the limited availability of dosimetric information in published studies, irradiance values can be challenging. While many studies do not report irradiance values, they can be calculated based on other provided parameters. A phenomenon known as “hormesis” exists, where very low doses of certain parameters, such as irradiance (mW/cm^2^), may result in insignificant therapeutic effects. Evidence suggests that an irradiance above 300 mW/cm^2^ is required to inhibit Aδ and C pain fibers when absorbed by tissue [12,33]. In this study, an irradiance of 333 mW/cm^2^ was selected based on findings suggesting significant differences between groups using irradiance levels greater than 300 mW/cm [13,29,34]. Future research in this area should avoid irradiance levels below 300 mW/cm^2^, as they may not achieve the desired therapeutic outcomes.

The exposure time per point is also an important factor. Incorrect timing may render PBM ineffective. A very short application time may not produce an effect, while excessive application times could cause inhibitory effects. In dentistry, treatment times vary between 30 and 60 s per point [5]. This parameter often varies widely between studies in the literature, with the shortest being 15 s [29] and the longest at 180 s [8]. This clinical trial used a time related to positive outcomes in other studies of post-endodontic pain [7,13]. Future studies should consider increasing the time and energy provided per point, associated with analyzing the preoperative bone structure of each participant with the assistance of cone beam computed tomography (CBCT).

How endodontists conduct root canal therapy is a key factor that can affect the severity of post-endodontic pain [3]. Despite using the reciprocating system, which is directly related to increased neuropeptides of C-type nerve fibers [3], this clinical trial demonstrated that the adoption of a well-defined endodontic treatment protocol, combined with properly performed crown-down preparation, emphasizing effective canal disinfection, and the experience of a single operator, results in low post-endodontic pain levels, with median scores around 0.2 cm on the Visual Analog Scale, as well as a significant reduction in the need for analgesic use.

Few clinical studies comprehensively assess pain, incorporating semiological maneuvers such as palpation and percussion. In this study, pain analysis was performed using palpation (buccal and palatal) and percussion (vertical and horizontal) maneuvers, with no differences found between the groups 24 h after endodontic treatment. Of the studies reviewed, only two included this analysis [11,35], and none found significant differences between the groups.

Currently, presenting an effect size measure to increase the significance of results in hypothesis testing has become important. Statistics can provide comparable effect size measures for studies using different outcome measures [34]. In this clinical trial, the explained proportional variance analysis showed the highest value for the primary outcome of pain at 24 h (η^2^ = 0.160) and the lowest for pain at 12 h (η^2^ = 0.000).

Although the observed effect size was relatively small, no prior studies were identified that reported effect sizes in a manner that would allow for direct comparison. It appears that the greatest effect size is associated with the endodontic treatment itself—the primary intervention for addressing the condition—while photobiomodulation (PBM) seems to play a more modest role. Future studies with greater statistical power—either through larger sample sizes or the use of more homogeneous subgroups (comprising individuals more likely to benefit from the intervention and thus present larger effect sizes)—may prove valuable. This approach would enable clearer identification of which experimental designs or subpopulations tend to exhibit more significant responses. Moreover, it is worth noting that in time-series analyses, effect sizes observed at individual time points may seem small. However, when considered cumulatively over time, these effects often become more substantial, as they better reflect the complex, multidimensional nature of pain [34].

No statistical differences were found between the groups in the OHIP-14 outcome (*p* = 0.347), indicating that the effects of endodontic treatment on participants’ quality of life were not influenced by PBM.

Current systematic reviews call for the inclusion of preoperative pain frequency in the data presented [5]. In this clinical trial, the preoperative pain frequency was 41.6% for the PBM group and 37.5% for the control group. The other pain frequencies found in the study, based on the Youden index for the different time intervals analyzed, ranged from 12.5% to 25%, corroborating with other studies in the literature [6].

Like this clinical trial, none of the studies reviewed reported adverse effects of low-level lasers [3].

The first limitation of this study was the inadequate analgesic use by six participants (four in the control group and two in the PBM group), due to fear of pain. Originally planned for analysis, the analgesic intake had to be excluded from the outcomes due to the small number of participants who used the medication and the improper way it was used. This is an important discussion present in all studies evaluating pain as an outcome. For ethical reasons, interfering with patient medication intake is not possible. Still, it is important to provide the best guidance to participants so they trust the treatment.

Measuring pain, such a subjective issue, is complex. Despite using the millimeter scale, a unidimensional instrument suited for pain studies [36], patient interpretation of the scale also posed a limiting factor, as some participants misused the scale or reduced their pain rating to zero for what they considered discomfort. Including a larger and more anatomically homogeneous sample would be a valuable recommendation for future studies, as it would enhance statistical power and improve the generalizability of the findings.

Although the 808 nm wavelength was selected based on previous evidence demonstrating its biological effects in reducing inflammatory mediators and nociceptive responses in endodontic applications [7,13], it is important to acknowledge the limited penetration capacity of near-infrared light through dense cortical bone. This optical attenuation represents a relevant limitation, particularly in maxillary molars, which are often surrounded by thick osseous structures. Future studies should investigate alternative wavelengths in conjunction with individualized CBCT-based assessments to tailor dosimetric parameters and improve light–tissue interaction in anatomically complex regions. This recommendation is consistent with recent findings by Henderson et al. 2024 [31], who emphasized the critical role of wavelength selection in optimizing light transmission through osseous tissues [31].

Finally, the study did not include long-term follow-up to focus on the critical window of post-endodontic pain as suggested by previous literature [6]. The findings indicated low pain levels following properly performed endodontic treatment. So, pain assessments beyond the 24 h period may offer limited additional insight. Interestingly, consider that the most relevant impact of PBM may occur precisely within this early time frame. Future studies could benefit from isolating this initial 4 h period to more accurately characterize the analgesic effects of PBM during the peak of post-treatment pain.

Subsequent research should aim to refine treatment through personalized dosimetry, taking into account the specific anatomical characteristics of the region or dental element under analysis. Parameters such as cortical bone thickness and the number of roots (apices), obtained via complementary imaging techniques like cone beam computed tomography (CBCT), may provide crucial data to support a more individualized therapeutic approach.

## 5. Conclusions

This randomized clinical trial demonstrated that PBM, when applied with the tested parameters and targeted at the maxillary bone, does not exert an analgesic effect on postoperative pain following endodontic treatment of maxillary molars within the first 24 h. Future investigations should prioritize personalized dosimetry based on the specific anatomical characteristics of the region or dental element under treatment—ideally guided by advanced imaging modalities such as CBCT—to enhance the precision and therapeutic efficacy of PBM protocols.

## Figures and Tables

**Table 1:** Dosimetric parameters.

Parameters	Values
Wavelength [nm]	808
Operating mode	Continuous
Power [mW]	100
Irradiance [mW/cm^2^]	333.3
Output area [cm^2^]	0.3
Exposure time [s] per point	30
Radiant exposure [J/cm^2^] per point	10
Energy [J] per point	3
Total energy [J]	9
Number of radiated points	3
Application	Contact
Application site	Apex Radicular
Number of sessions	Single

nm—nanometers, mW—milliwatts, W/cm^2^—watts per square centimeter, s—seconds, J/cm^2^—Joules per square centimeter.

**Table 2:** Demographic and clinical variables according to the groups.

Variable	Control	PBM	*p*
	*n* = 24	*n* = 24	
Age (years), mean and SD	40.75 ± 13.310	41.83 ± 10.54	0.756 ^a^
Gender—Female, % Male, %	17 (70.84%) 7 (29.16%)	12 (50%) 12 (50%)	0.140 ^b^
Treated tooth			0.074 ^b^
1st maxillary molar	18 (75%)	12 (50%)	
2nd maxillary molar	6 (25%)	12 (50%)	

^a^: Student’s *t*-test, ^b^: Pearson’s chi-square test; SD: standard deviation; PBM: photobiomodulation.

**Table 3:** Postoperative pain (cm VAS) at 4, 8, 12, and 24 h in the PBM and control groups. Data are presented as median (interquartile range).

Variable	Control	PBM	*p*	η^2^
	*n* = 24	*n* = 24		
Pain at 24 h	0.15 (0.1; 0.2)	0.2 (0.1; 0.2)	0.515 ^d^	0.160
Pain at 4 h	0.2 (0.1; 0.35)	0.2 (0.1; 0.2)	0.774 ^d^	0.103
Pain at 8 h	0.2 (0.1; 0.275)	0.2 (0.1; 0.2)	0.887 ^d^	0.050
Pain at 12 h	0.2 (0.1; 0.2)	0.2 (0.1; 0.2)	0.772 ^d^	0.000
Pain on vestibular palpation (24 h)	0.2 (0.1; 1.4)	0.2 (0.1; 1.975)	0.651 ^d^	0.045
Pain on palatal palpation (24 h)	0.15 (0.1; 0.2)	0.2 (0.1; 0.2)	0.686 ^d^	0.086
Pain on vertical percussion (24 h)	0.2 (0.1; 0.35)	0.2 (0.1; 1.5)	0.704 ^d^	0.047
Pain on horizontal percussion (24 h)	0.2 (0.1; 1.775)	0.2 (0.1; 1.9)	0.974 ^d^	0.044

^d^: Mann–Whitney U test; PBM: photobiomodulation.

**Table 4:** OHIP-14: Analysis between groups. Data are presented as median (interquartile range).

Variable	Control	PBM	*p*
	*n* = 24	*n* = 24	
OHIP 14	10.5 (2.0; 19.75)	12.0 (5.25; 18.5)	0.347 ^d^

^d^: Mann–Whitney U test; PBM: photobiomodulation.

**Table 5:** Pain frequencies at the intervals of preoperative, 4 h, 8 h, 12 h, and 24 h according to the evaluated groups.

Variable	Control	PBM	*p*
	*n* = 24	*n* = 24	
Preoperative pain frequency	9/24 (37.5%)	10/24 (41.6%)	0.768 ^e^
Pain frequency at 4 h	6/24 (25%)	4/24 (16.6%)	0.477 ^e^
Pain frequency at 8 h	6/24 (25%)	5/24 (20.8%)	0.731 ^e^
Pain frequency at 12 h	4/24 (16.6%)	4/24 (16.6%)	1.000 ^e^
Pain frequency at 24 h	3/24 (12.5%)	6/24 (25%)	0.267 ^e^

^e^: Kappa coefficient; PBM: photobiomodulation.

## Data Availability

All data will be available for the readers.
